# Rare-earth based materials: an effective toolbox for brain imaging, therapy, monitoring and neuromodulation

**DOI:** 10.1038/s41377-022-00864-y

**Published:** 2022-06-10

**Authors:** Zheng Wei, Yawei Liu, Bo Li, Jingjing Li, Shuang Lu, Xiwen Xing, Kai Liu, Fan Wang, Hongjie Zhang

**Affiliations:** 1 State Key Laboratory of Rare Earth Resource Utilization, Changchun Institute of Applied Chemistry, Chinese Academy of Sciences, Changchun, 130022 China; 2 School of Applied Chemistry and Engineering, University of Science and Technology of China, Hefei, 230026 China; 3 Department of Biotechnology, College of Life Science and Technology, Jinan University, Guangzhou, 510632 China; 4 Department of Chemistry, Tsinghua University, Beijing, 100084 China

**Keywords:** Optical materials and structures, Imaging and sensing

## Abstract

Brain diseases, including tumors and neurodegenerative disorders, are among the most serious health problems. Non-invasively high-resolution imaging methods are required to gain anatomical structures and information of the brain. In addition, efficient diagnosis technology is also needed to treat brain disease. Rare-earth based materials possess unique optical properties, superior magnetism, and high X-ray absorption abilities, enabling high-resolution imaging of the brain through magnetic resonance imaging, computed tomography imaging, and fluorescence imaging technologies. In addition, rare-earth based materials can be used to detect, treat, and regulate of brain diseases through fine modulation of their structures and functions. Importantly, rare-earth based materials coupled with biomolecules such as antibodies, peptides, and drugs can overcome the blood-brain barrier and be used for targeted treatment. Herein, this review highlights the rational design and application of rare-earth based materials in brain imaging, therapy, monitoring, and neuromodulation. Furthermore, the development prospect of rare-earth based materials is briefly introduced.

## Introduction

Brain diseases are globally challenging issues owing to the special lesion sites, complicated pathogenesis, high mortality, and poor prognosis^1–4^. However, the complexity of the cerebral nerves, the limited regenerative capacity of brain tissues, and difficulty in delivering conventional drugs through the blood-brain barrier (BBB), severely restrict the diagnosis and treatment of brain diseases^5,6^. Moreover, the brain is fragile, and minor disorders and trauma could lead to severe dysfunction. Therefore, the development of non-invasive, rapid, and efficient visual diagnosis and treatment techniques is essential for understanding and treating brain diseases. Especially, the combination of imaging and therapy can greatly simplify the treatment process of brain diseases and reduce damage to brain tissue. Current methods of brain imaging, such as magnetic resonance imaging (MRI), fluorescence imaging and computed tomography (CT), are limited with biosafety, sensitivity, penetration depth, and resolution. In addition, craniotomy is usually required for brain treatment. Therefore, it is important to design new imaging and therapeutic agents to address brain diseases in a gentle and valid way.

Among the imaging and therapeutic agents, rare-earth based materials show great advantages and application prospects in brain imaging and brain diseases treatment due to their unique electrical, optical and magnetic properties^7–10^. Materials doped with Gd^3+^, Ho^3+^ and Dy^3+^ ions possess high magnetic moment, high X-ray absorption coefficients and relatively long electron relaxation time^11–13^, enabling high spatial resolution of *T*_1_ -weighted, *T*_2_ -weighted MRI and CT brain imaging. Moreover, the emission spectrum of the lanthanide (Ln) based materials could be modulated from ultraviolet (UV) region to near-infrared (NIR) region by excitation with NIR source, which could be used for down-conversion (DC) and upconversion luminescence (UCL) imaging^14^. Importantly, NIR light can prevent damage to normal tissues and avoid background fluorescence of biological systems, enabling brain imaging under the skull. Particularly, Ln^3+^ ions with different properties can be synthesized and doped into a single nanomaterial to realize multi-modal imaging, providing a powerful tool for high-quality imaging of the brain^15^. In addition to brain imaging, rare earth-based nanoparticles (RENPs) are available for image-guided synergistic therapy of brain diseases^16^. Various therapeutic strategies based on RENPs such as radiotherapy, photodynamic therapy (PDT) and photothermal therapy (PTT) have been extensively studied and reported^17–21^. Moreover, by detecting the fluorescence changes, RENPs are also effective in monitoring neuronal activity, sensing ionic changes in the brain, and visualizing brain diseases^22–24^. Furthermore, the RENPs-mediated wireless optogenetics technique is also developed for manipulating neural activity and treating neurological disorders^25^. Importantly, the functionalization of RENPs can further improve their stability, biocompatibility, targeting ability and the ability to overcome the BBB^26–29^. Therefore, the rare-earth based materials are expected to break through the current limitations of brain diseases imaging, therapy and early diagnosis.

**Fig. 1:**
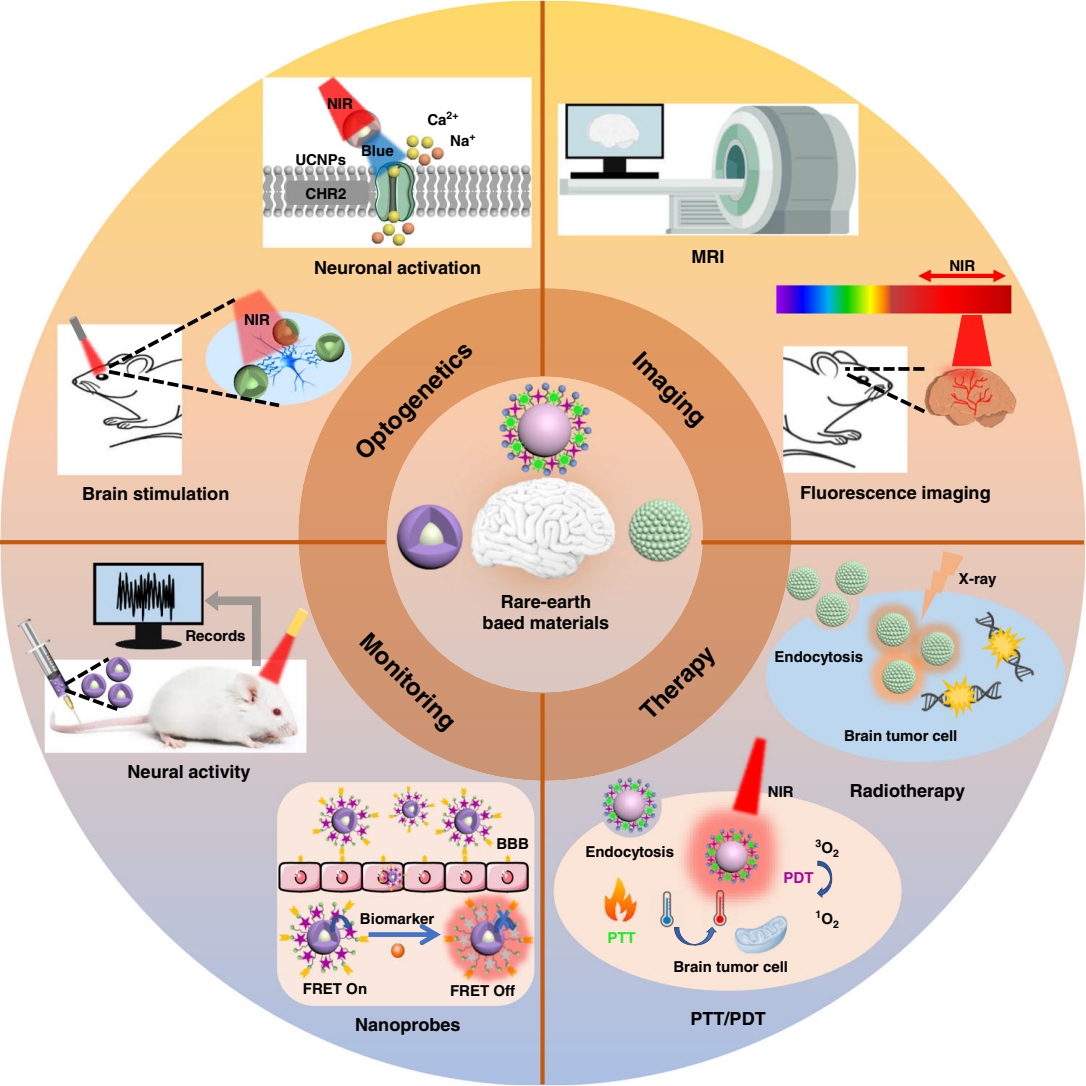
The overview of applications of rare-earth based materials for brain disease diagnosis and treatments

Herein, the recent research in the preparation and application of rare earth-based diagnostic and therapeutic materials are summarized. We highlight four applications of rare-earth based materials, including brain imaging, brain diseases therapy, brain disease diagnosis and monitoring, and brain modulation through optogenetics (Fig. 1). The functional properties and structural design of rare-earth based materials are discussed in detail. The prospects and potential challenges of rare earth-based materials in brain-related clinical applications are also presented.

## Rare-earth based materials for brain imaging

### Magnetic resonance imaging

MRI has emerged as a safe, painless, and powerful diagnostic tool, which is widely used in brain imaging^30^. High spatial resolution brain images are conducive to visualizing the mass lesions in brain tumors^31,32^. In the process, contrast agents (CAs) are indispensable for obtaining high signal-to-noise ratio images^33^. Among them, rare-earth based composites with superior optical and magnetic properties attract great attention due to their unique 4f external electronic structure. In particular, Gd^3+^ ions can provide a high electron magnetic moment and effectively shorten the electron relaxation time because of the seven unpaired 4f electrons (^8^S_7/2_) and symmetrical ground state^34,35^.

Currently, Gd-based complexes, for example, Gd-diethyltriaminepentaacetic acid (Gd-DPTA), are used as clinical *T*_*1*_ -weighted MRI contrast agents. They exhibited high sensitivity in monitoring BBB rupture, blood vessel, and hemodynamics^36^. However, owing to the low longitudinal relaxivity (*r*_*1*_ = 3.5 mM^−1^ s^−1^) and short half-life (20 min), Gd-DPTA displayed poor selectivity to tissues and limited time-dependent brain imaging property^37^. To address these problems, Li et al. embedded Gd-DPTA into Poly-N^5^-(3-hydroxypropyl)-L-glutamine (PHTG) chains to restrict rotational movement of Gd-DTPA moieties, and developed a new biodegradable nonionic polymeric CA, named PHPG-DTPA-Gd^38^. It exhibited 3.7 times higher longitudinal relaxivity than Gd-DPTA. Notably, the terminal elimination half-life of PHPG-DTPA-Gd was extended to 8.4 h, allowing for long-term imaging. Benefiting from the long half-time and high *r*_*1*_ value, cerebral vessels in mice could be imaged for 2 h with high resolution. Importantly, the PHPG-DTPA-Gd demonstrated superior biocompatibility and biodegradability due to their intrinsic nature. NaGdF_4_ nanoparticles with high loading of Gd^3+^ are also effective MRI CAs. The longitudinal relaxation rate of NaGdF_4_ is (*r*_*1*_ = 8.93 mM^−1^ s^−1^) about 2.3 times higher than that of conventional clinical CA (*r*_*1*_ = 3.8 mM^−1^ s^−1^)^39^. Yao et al. used HIV-1 transactivator (TAT) peptides-modified NaGdF_4_ (NaGdF_4_-TAT) to successfully track the fate of adoptive T-cells in an orthotopic glioblastoma bearing mice by *T*_1_-weighted MRI^40^.

**Fig. 2:**
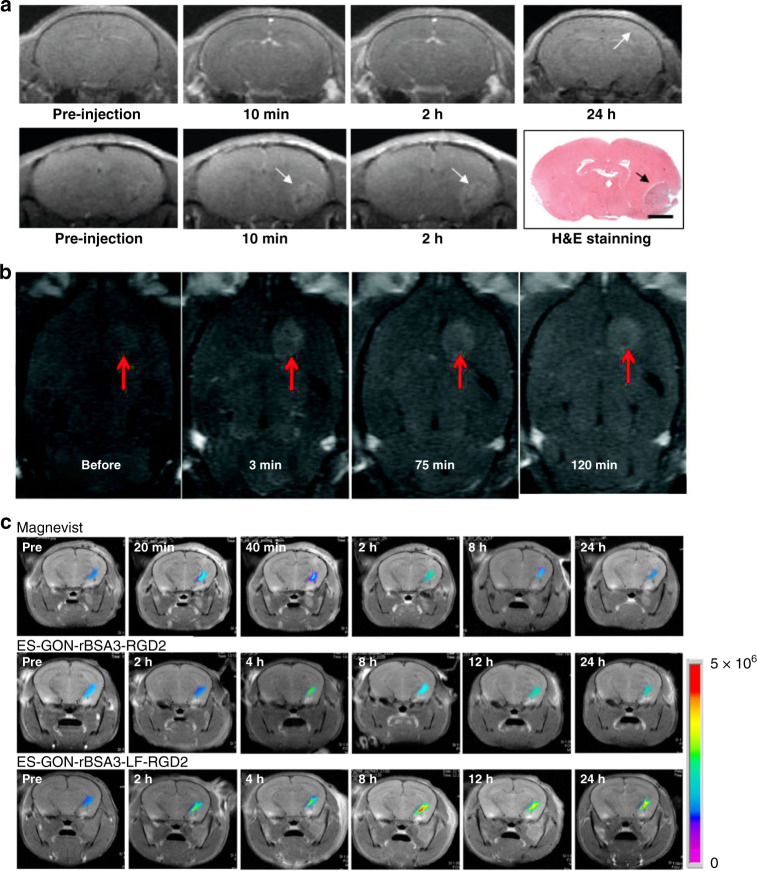
In vivo brain MRI by using Gd-based CAs. **a** In vivo MRI of normal brain (top) and tumor-bearing brain (bottom) before and after injection of Den-RGD-Angio at different times. Reprinted with permission from ref. ^41^. Copyright 2012 American Chemical Society. **b** In vivo MRI of rat brain tumor after injection of ultra-small Gd_2_O_3_ nanoparticles. Reprinted with permission from ref. ^45^. Copyright 2009 American Chemical Society. **c** In vivo MRI of tumor-bearing brain after injection of Magnevist, ES-GON-rBSA3-RGD2, and ESGON-rBSA3-LF-RGD2 at different times. Reprinted with permission from ref. ^47^. Copyright 2020 Elsevier

In addition to the short circulation lifetime in vivo, non-target specificity and poor BBB permeability are still challenging for the clinical applications of Gd-based CAs. To overcome these challenges, Li and coworkers designed a novel two-order targeted nanoprobe (Den-RGD-Angio)^41^. The fifth generation (G5) polyamide-amine (PAMAM) dendrimer was conjugated with DOTA moieties to chelate Gd^3+^ ions for high contrast MRI (Fig. 2a). Subsequently, cyclic (RGDyK) peptides and angiopep-2 peptides were grafted to the chain of PAMAM dendrimer to realize tumor targeting and effective penetration of BBB, respectively^42,43^. Furthermore, the size of the Gd-based nanoparticles was proved to affect brain imaging quality^44^. The high proportion of surface Gd^3+^ ions in ultra-small nanoparticles greatly induces the longitudinal relaxation of water protons through synergistic effect of Gd^3+^ ions, which improves the imaging contrast significantly (Fig. 2b)^45^. Moreover, various strategies were applied to improve the biocompatibility of Gd-based CAs. Fortin et al. prepared one-pot PEG-coated Gd_2_O_3_ nanoparticles to improve the biocompatibility of Gd_2_O_3_ nanoparticles^46^. Chen et al. prepared poly (acrylic acid) (PAA)-stabilized Gd_2_O_3_ nanoparticles in aqueous phase and modified them with reduced bovine serum albumin (rBSA) to achieve good biocompatibility^47–49^. Moreover, RGD dimer and lactoferrin (LF) were further covalently grafted to the Gd_2_O_3_ nanoparticle to achieve the BBB targeting and penetration. The achieved ES-GON-rBSA3-LF-RGD2 nanoparticles displayed an *r*_*1*_ of 60.8 mM^−1^ s^−1^ at 1.5 T. After 12 h’ injection of nanoparticles, the signal of *T*_*1*_-weighted MRI was enhanced to 423 ± 42%, which was 5–6 times higher than that of commercial Gd-based CAs (<80%), enabling high-contrast MRI of orthotopic glioblastoma (Fig. 2c).

### Fluorescence imaging

Fluorescence imaging possesses the advantages of non-radiotoxicity, non-invasive, rapid detection, high sensitivity and superior imaging resolution^50–52^. Over the past few decades, the fluorescence imaging mainly focused on the visible region (400–700 nm) and NIR-I window (700–900 nm). However, photon scattering or photon absorption always occur when light enters tissue or bone, resulting in inevitable thermal damage, limited penetration depth and low signal-to-noise ratio^51^. Craniotomy, bone window opening and cranial grinding processes are usually required for traditional fluorescence imaging, further leading to damage to brain tissue and cerebral vessels^5^. The NIR-II (1000–1700 nm) fluorescence imaging technology is proved to have a higher spatial resolution, less thermal damage, deeper penetration depth and lower tissue autofluorescence. It can reach a depth of millimeters at sub-10 μm resolution to visualize the brain in real-time without the need for craniotomy^53^. As promising nanomaterials for the next generation of in vivo fluorescence imaging, RENPs have superior photostability, long fluorescence lifetimes, narrow emission bandwidths and high biocompatibility^14,54^. Particularly, their rich energy level transitions allow for the tunable NIR-II emission by changing the doped ion species^50^. Table 1 summarizes typical rare-earth based materials for NIR-II fluorescence imaging.

**Table 1:** Typical rare-earth based materials for NIR-II fluorescence imaging

Rare-earth based materials	Excitation wavelength (nm)	Emission wavelength (nm)	NIR-II quantum yield (%)	Penetration depth (mm)	Year
NaYbF_4_:Er/Ce @NaYF_4_	980	1550	2.73	>1.3 (under the intact scalp and skull)	2017^53^
NaLnF_4_:Gd/Yb /Er/Ce/nanorod	980	1525	3.6	–	2019^55^
NaErF_4_:Yb@ NaLuF_4_	808	1525	11	–	2018^56^
Hexagonal-phase NaErF_4_@NaYF_4_	808	1525	10.2	–	2020^57^
NaYF_4_:Yb:Nd@CaF_2_	800	1000	11	–	2018^58^
NaErF_4_:Tm@ NaLuF_4_	800	1525	13.92	–	2021^59^
KSc_2_F_7_:Yb^3+^/Er^3+^	980	1525	2.7	–	2018^60^
NaNdF_4_@NaLuF_4_/IR-808	808	1060/1340	–	–	2019^61^
RENPs@Alk-Pi	808	1525	–	–	2021^62^
NaErF_4_:Ce@ NaYbF_4_@NaLuF_4_-Dye-BP	808	1525	–	>3	2020^63^
QDs-LnNCs	975	1550	0.25	–	2021^64^
Er(III)–bacteriochlorin complexes	760	1530	0.01(absolute quantum yield)	2 (in the hippocampus)	2021^65^

**Fig. 3:**
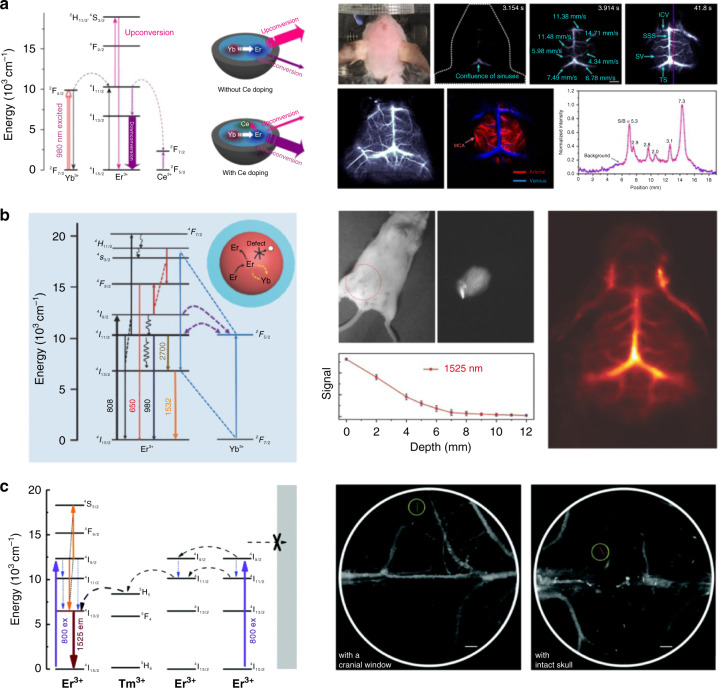
Intravital fluorescence imaging of cerebrovascular structure by using different RENPs. **a** Schematic diagram of the energy-transfer process of Er-based RENPs doped with and without Ce^3+^ ions (left). Fluorescence images of cerebral vessels with Ce^3+^ doped Er-RENPs (right). Reprinted with permission from ref. ^53^. Copyright 2017 Yeteng Zhong et al. **b** Energy level diagrams of the self-sensitized nanoparticles (left). Intravital fluorescence imaging of cerebral vessels after injection of NaErF_4_:Yb^3+^@NaLuF_4_ core/shell nanocrystals (right). Reprinted with permission from ref. ^56^. Copyright 2018 Wiley-VCH GmbH. **c** Energy-transfer mechanisms of the NaErF_4_:Tm^3+^@NaLuF_4_ core-shell nanoparticles (left). The cerebrovascular images of the mice with a cranial window and with an intact skull under NIR excitation (right). Reprinted with permission from ref. ^59^. Copyright 2021 The Royal Society of Chemistry

Er^3+^-doped RENPs can emit luminescence at around 1500 nm, which are the most widely reported NIR-II imaging reagents. Dai’s group reported a bright 1550 nm luminescent Er-based nanoparticle under 980 nm excitation^53^. Ce^3+^ ions were co-doped into NaYbF_4_ nanocrystal to suppress the upconversion process and promote 1550 nm emission by accelerating the effective photon-assisted nonradiative cross relaxation between Ce^3+^ ions and Er^3+^ ions^66^. Moreover, the inert NaYF_4_ shell was applied to protect Er^3+^ ions from surface quenching^67^. In addition, stability and biocompatibility were greatly enhanced by surface modification of amphiphilic polymer and PEG. Fast in vivo imaging of mice’s cerebrovascular structure at a depth of 1.3 mm with an exposure time of only 20 ms was achieved by using this bright NIR-II fluorescent probe (Fig. 3a). Hao’s group also fabricated the PAA-modified NaLuF_4_:Gd/Yb/Er/Ce nanorods for cerebrovascular imaging^55^. The emission of RENPs beyond 1500 nm in an aqueous solution was enhanced by 1.75 to 2.2 times with co-doped with Ce^3+^ ions. Besides, the quantum yield of RENPs increased from 2.2% to 3.6% by replacing the NaYF_4_ host with NaLuF_4_ host. After 8 s injection of RENPs, the cerebral vessels of mice could be visualized clearly. Additionally, Chen’s group reported a novel Er^3+^ self-sensitized core-shell NaErF_4_:Yb^3+^@NaLuF_4_ under 800 nm excitation^56^. The inert shell of NaLuF_4_ inhibited the luminescence concentration quenching and surface quenching, allowing 100% incorporation of Er^3+^ ions into the core. In addition, Yb^3+^ ions were doped as the energy trapping centers for enhancing NIR-II emission (Fig. 3b). The obtained RENPs possessed a high quantum yield of about 11%, enabling transcranial imaging of cerebral vessels in mice with an exposure time of 10 ms.

Furthermore, Chen’s group investigated the influence of crystal phase, core-shell structure, shell matrix and shell thickness on the NIR-II emission of Er^3+^ self-sensitized nanoparticles^57^. The brightest emission was obtained by hexagonal (β)-phase NaErF_4_@NaYF_4_ nanoparticles with an ultra-small size of 13 nm. Importantly, owing to the ultra-small size, these nanoparticles were completely excreted through feces in 14 days. Meanwhile, Liu et al. developed an efficient Er^3+^ self-sensitized system of NaErF_4_:Tm^3+^@NaLuF_4_^59^. Tm^3+^ ions were introduced as the energy capture center and facilitated the energy transfer between Tm^3+^ ions and Er^3+^ ions. The obtained NaErF_4_:Tm^3+^@NaLuF_4_ nanoparticles exhibited a high quantum yield of 13.92%. After further modification with hydrophilic PEG, the nanoparticles achieved accurate NIR-II images of cerebral vessels with an intact skull (Fig. 3c). In addition to the Ln-based host, a new orthorhombic Sc-based nanoprobe that co-doped with Yb^3+^/Er^3+^ was developed by Liu et al.^60^. The Sc-based nanoprobes showed outstanding NIR-II luminescence at 1525 nm with a quantum yield of 2.7%, which could support non-invasive cerebrovascular visualization.

**Fig. 4:**
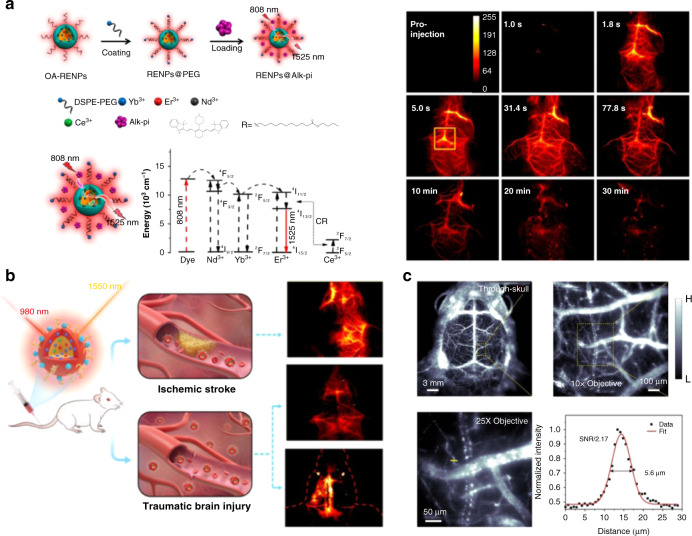
Different sensitization strategies to improve the luminescent brightness of RENPs for fluorescence imaging in the brain. **a** Schematic diagram of constructing dye-sensitized RENPs (left). Fluorescence imaging of brain vasculature after injection of RENPs@Alk-pi under 808 nm excitation (right). Reprinted with permission from ref. ^62^. Copyright 2021 American Chemical Society. **b** QDs-sensitized RENPs for high-contrast of dynamic cerebrovascular changes in traumatic brain injury. Reprinted with permission from ref. ^64^. Copyright 2021 American Chemical Society. **c** Bacteriochlorin-sensitized RENPs for intravital microscopic imaging of the cerebral vessels. Reprinted with permission from ref. ^65^. Copyright 2021 Nature Publishing Group

Although RENPs are desirable CAs for brain imaging, the absorption cross sections of the Ln^3+^ ions are extremely small, which impairs their performance in bio-imaging^54^. The absorption cross sections of organic dyes are 10^3^–10^4^ times larger than Ln^3+^ ions, which can be employed as antennas to improve the light-harvesting capacity of Ln^3+^-based nanocrystals^68–70^. Li et al. electrostatically anchored IR-808 molecules onto NaNdF_4_@NaLuF_4_ nanoparticles within a spatial distance of 2.4 nm, allowing the occurrence of Förster resonance energy transfer (FRET) from IR808 to Nd^3+^ ions^61^. By synergistically absorbing 808 nm light and efficiently transferring excited state energy, the NIR-II emission at 1060 nm and 1340 nm was significantly enhanced. The DSPE-PEG_5000_ was further coated to improve biocompatibility of nanoparticles. Finally, orthotopic glioblastoma was detected clearly by NIR-II fluorescence imaging with the focused ultrasound (FUS)-assisted technique^71^. In another work, Liu’s group designed new NIR dyes to sensitize NaYF_4_:Yb/Er@NaYF_4_:Nd nanoparticles via the energy-transfer cascade: dye→Nd^3+^→Yb^3+^→Er^3+^ (Fig. 4a)^62^. They optimized the structure of NIR dyes by introducing long hydrophobic alkyl chains and electron-donating group. The as-obtained Alk-pi modified RENPs achieved a 40-fold enhancement of the NIR-II luminescence, providing high-resolution NIR imaging of mice’ brains without the need for craniotomy or skull thinning. To increase the stability of dye, Ren et al. designed a new dye-brush polymer (Dye-BP) and applied it on the surface of Er-based down-conversion nanoparticles (DCNPs) through ligand exchange processes^63^. Through co-harvesting 808 nm photons by Dye-BP and transferring photons to ^4^I_9/2_ energy level of Er^3+^ ions, the NIR-II emission of Er-DCNPs-Dye-BP was enhanced by 675-folds. Other materials with large absorption cross sections can also be applied as an antenna to enhance the luminescent intensity of RENPs^54^. The extinction coefficient of Ag_2_Se quantum dots (QDs) is 2.8 L g^−1^ cm^−1^, which was 530 times higher than that of Yb-RENPs (5.2 × 10^−3^ L g^−1^ cm^−1^)^64,72^. The Ag_2_Se QDs sensitized RENPs possessed a 100-fold enhancement in the NIR-II luminescence intensity, enabling clear observation of dynamic cerebrovascular changes in traumatic brain injury (Fig. 4b). Inspired by the light-harvesting role of bacteriochlorin, Zhang et al. proposed a novel bacteriochlorin-sensitized NIR probe (EB766)^65^. Bacteriochlorin acted as the antenna ligand to populate photons at the ^4^I_13/2_ level of Er^3+^ with an efficiency of about 99.9% and a rate constant of 2 × 10^9^ s^−1^. BSA and cell-penetrating peptide HIV-TAT were further added to label the cancer cells for non-invasively monitoring their movement, migration, and residence in the cerebrovascular wall of the mouse through intravital NIR-II imaging (Fig. 4c).

### Multi-modal imaging

**Fig. 5:**
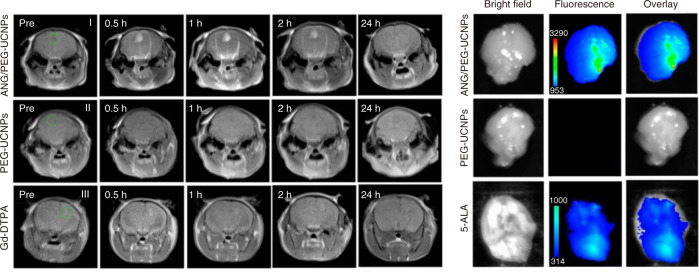
Intravital dual-modal MRI/UCL imaging of brain tumor after injection of ANG/PEG-UCNPs, PEG-UCNPs and Gd-DTPA. Reprinted with permission from ref. ^76^. Copyright 2014 American Chemical Society

MRI and fluorescence imaging are powerful tools for brain imaging. However, MRI displays poor spatial resolution, while fluorescence imaging has low fluorescence quantum yield^73^. To address these shortcomings, multi-modal imaging was developed to provide more accurate imaging information of brain for further clinical applications. Notably, Ln^3+^ ions possess superior optical, magnetic properties, and high X-ray absorption coefficients (2.32–4.01 cm^2^ g^−1^), allowing for simultaneous MRI, fluorescence imaging and CT imaging on a single material^13,73,74^. For example, Wong’s group integrated Gd^3+^-DOTA and NaYF_4_:Yb/Er/Tm upconversion nanoparticles (UCNPs) for MR/ UCL dual-modal imaging of glioblastoma^75^. The conjugated UCNP-Gd reagents displayed a high *r*_*1*_ of 12.741 mM^−1^ s^−1^ at 300 MHz and strong UCL in deep-red region. RGD was further modified on the UCNP-Gd to specifically identify U87MG cells. As a result, the boundaries of glioblastoma could be delineated preoperatively and the resection of glioblastoma could be guided by the MRI/UCL imaging. Recently, Shi’s group realized simultaneously MRI/fluorescence imaging on the single Gd-doped NaYF_4_:Yb/Tm/Gd@NaGdF_4_ nanoparticle (Fig. 5)^76^. The introduction of Gd^3+^ ions provides high contrast MRI. Meanwhile, Tm^3+^ ions show strong UCL under 980 nm excitation. PEG and angiopep-2 peptide were then coupled onto NaYF_4_:Yb/Tm/Gd@NaGdF_4_ nanoparticles for water solubility and effective penetration of the BBB, respectively. These nanoprobes achieved preoperative diagnosis and intraoperative localization of glioblastoma, which are better than the clinical MRI CA (Gd-DTPA) and fluorescent dye (5-ALA). In another exciting work, they prepared Ho^3+^-doped NaYbF_4_ UCNPs for tri-modal MRI/UCL/CT imaging^77^. The short electronic transverse relaxation time and high effective magnetic moments of Ho^3+^ ions and Yb^3+^ ions made the UCNPs suitable for *T*_*2*_ -weighted MRI^12,35^. In addition, Ho^3+^-based NaYbF_4_ UCNPs could emit 540 nm light under 980 nm excitation, allowing for UCL imaging. Moreover, Ho^3+^ and Yb^3+^ ions possess high X-ray absorption coefficients, which are suitable for CT imaging. Therefore, MRI/UCL/CT tri-modal imaging could be achieved in single Ho^3+^-based NaYbF_4_ UCNPs. Additionally, Lu-based RENPs show the best CT imaging quality due to the high X-ray absorption coefficient of Lu^3+^ ions (4.01 cm^2^ g^−1^ at 100 keV). Zhou et al. prepared CsLu_2_F_7_:Yb/Er/Tm-based nanoparticles for CT/UCL dual-modal imaging^78^. Heavy alkali metal of Cs was used to replace Na in the UCNPs host lattice to provide enhanced CT signals. As a result, the dual-modal of CT and optical imaging realized real-time imaging and precise diagnosis of brain tumor.

## Rare-earth based materials for brain disease therapy

### Radiotherapy

As the emerging multifunctional diagnostic and therapeutic agents, rare-earth based materials can be used not only for brain imaging but also for brain diseases therapy. Radiotherapy is a traditional treatment that uses radiation to locally kill tumors^79^. However, radiotherapy is insensitive to the S-phase cells and hypoxic cells within the tumor. High radiation doses are always required to eliminate tumor cells, which inevitably cause damage to nearby normal tissues. Gd-based therapeutic agents with a high X-ray absorption coefficient of 3.109 cm^2^ g^−1^ are attractive agents for radiotherapy, which can increase the deposition of local radiation dose at the tumor site and significantly enhance the therapeutic effect^47^. Olivier et al. proposed an imaging-guided radiotherapy through rapidly and precisely localizing Gd-based nanoparticles in the brain^17^. The superior MRI characteristics of Gd-based nanoparticles were used to monitor the real-time distribution of CAs in vivo. The irradiation only could be triggered when Gd-based nanoparticles were enriched in tumors, thereby significantly reducing the damage to surrounding healthy tissue. After 20 min of injection, more gadolinium was deposited in the diseased hemisphere, and the mice treated at this time displayed a long average survival time of 90 days.

### Photodynamic therapy

**Fig. 6:**
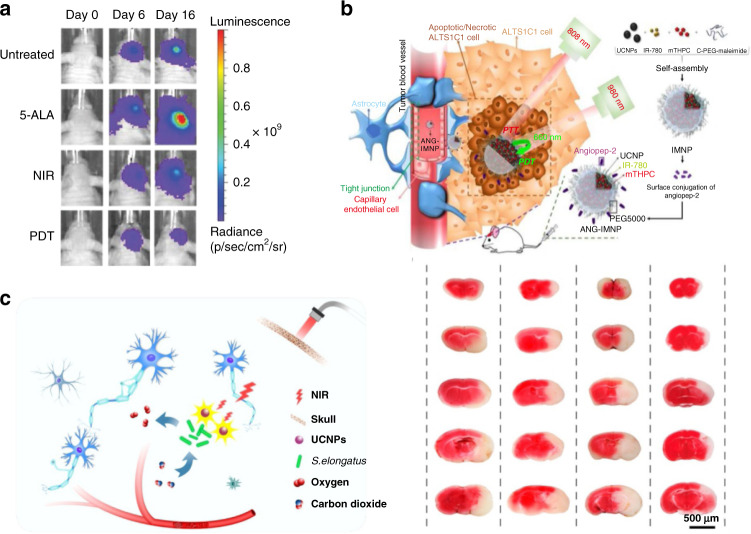
Rare-earth based materials for brain disease therapy. **a** PDT therapy of brain glioblastoma. Reprinted with permission from ref. ^18^. Copyright 2020 Wiley-VCH GmbH. **b** PDT/PTT synergistic treatment of brain glioblastoma. Reprinted with permission from ref. ^21^. Copyright 2018 Yuan-Chung Tsai et al. **c** The proposed mechanism of the NIR-light triggered NPT biosystem consisting of UCNPs and *S. elongatus* to treat ischemic stroke (left). The cerebral infarct volumes of brain in the stroke mice model with different treatments (right). Reprinted with permission from ref. ^19^. Copyright 2021 American Chemical Society

PDT is a powerful way to treat diseases, which eliminates tumor by reactive oxygen species (ROS) generated through the reaction of photosensitizers with oxygen under the irradiation^80^. Nevertheless, lots of photosensitizer molecules need to be excited by the UV or visible light that hardly penetrates the deep tissues and brain skeleton. The RENPs could emit tunable luminescence to sensitize the photosensitizers under NIR excitation, which greatly improve the penetration depth of PDT. Hyeon et al. assembled the photosensitizer Ce6 onto UCNPs via covalent conjugation and hydrophobic interaction for PDT treatment^81^. In this system, the red emission of UCNPs overlapped the absorption of Ce6, thereby inducing the PDT process under NIR excitation. In addition, UCNPs also acted as the carriers to deliver hydrophobic Ce6, which eliminates the aggregation of photosensitizers. Under 980 nm laser irradiation, the growth of U87MG tumor could be inhibited effectively. For enrichment of UCNPs at the tumor site along with rapid clearance at the end of treatment, Kennedy’s group designed an UCNP-based implant consisting of poly (ethylene glycol) diacrylate (PEGDA) core and fluorinated ethylene propylene (FEP) shell^18^. The photosensitizer precursor 5-ALA, which converted into the photosensitized metabolite protoporphyrin-IX (PpIX) in glioma cells, was pre-operatively injected to produce various ROS under the photoexcitation of implant^82,83^. Under 980 nm laser irradiation, cellular mitochondria in tumor cells were damaged and eventually led to the death of tumor cells (Fig. 6a). Apart from brain tumors, other forms of brain diseases can be treated by PDT. C_60_ molecule is a kind of functional photosensitizer molecule, which can jump to the triplet excitation state and generate ROS under UV or visible light. It can effectively scavenge ROS in the dark state owing to the huge unsaturated structure and superior electron affinity^84,85^. Based on this photo controlling mechanism, Qu et al. covalently linked C_60_ and amyloid-β (Aβ) targeting peptides with UCNPs (UCNP@C_60_-pep) to synergistically treat Alzheimer’s disease (AD)^20^. ROS generated by UCNP@C_60_-pep could specifically oxidize Aβ and increase the hydrophilicity of Aβ, thereby significantly reducing the aggregation of oxidized Aβ. In dark conditions, UCNP@C_60_-pep could scavenge overproduced ROS and maintain redox homeostasis in vivo. The dual properties of UCNP@C_60_-pep that either produce or scavenge ROS mitigated neurotoxicity, which could be used for alleviating Aβ-triggered paralysis and movement disorders.

### Photothermal therapy

PTT is another phototherapeutic strategy, which employs photothermal materials to convert NIR laser into heat to treat disease^86–89^. The high temperature can eliminate tumors by melting the membrane of tumor cells and denaturing proteins^90,91^. Chiu et al. integrated the photothermal molecule and photosensitizer onto a single UCNP to achieve synergistic PTT and PDT treatment of glioblastoma (Fig. 6b)^21^. Cellular experiments demonstrated that synergistic therapy could effectively ablate 82% of tumor cells. The median survival time of mice receiving the synergistic therapy was significantly prolonged to 24 days, which was 3 times longer than the control group. In another work, Qu et al. proposed a NIR-light controlled microglia activation strategy for targeted therapy of brain diseases. They prepared mesoporous silica coated UCNP as drug loading platforms^92^. Photothermal agents indocyanine green (ICG) and microglia activator-bacterial lipopolysaccharide (LPS) were loaded into the pores of SiO_2_. Subsequently, *β*-cyclodextrin (CD) and photoswitchable azobenzene (Azo) were further capped on the surface to control the release of drugs. The heat generated by the loaded ICG under irradiation could induce the expression of heat shock proteins in microglia, which synergistically enhanced the activation effect from LPS. As a result, the secretion level of pro-inflammatory cytokine in microglia was effectively promoted under NIR irradiation.

### Other therapies

In addition to radiotherapy, PDT and PTT, other new strategies have also been applied to synergistically treat brain disease with rare-earth based materials. Wang’s group developed a NIR-light triggered nanophotosynthetic (NPT) biosystem consisting of core-shell Nd^3+^-doped UCNPs and photoautotroph cyanobacterium (*S. elongatus*) to treat ischemic stroke (Fig. 6c)^19^. The depletion of oxygen as well as massive accumulation of carbon dioxide ultimately triggers irreversible neuronal damage. *S. elongatus* can spontaneously produce oxygen and consume carbon dioxide in the light. In vitro results showed that even in the presence of skull, UCNPs could drive *S. elongatus* to produce more oxygen in the NIR light (170.0 mmHg) than under dark conditions (152.6 mmHg). Therefore, the NPT biosystem could effectively protect neurons from hypoxia-induced injury. The hypoxic area and cerebral infarction volume of NPT-treated mice were reduced by 60.2% and 51.6%, respectively. Notably, NPT could effectively improve motor coordination and promote poststroke angiogenesis in stroke mice.

## Rare-earth based materials for early brain diseases monitoring and diagnosis

### Monitor the neural activity

**Fig. 7:**
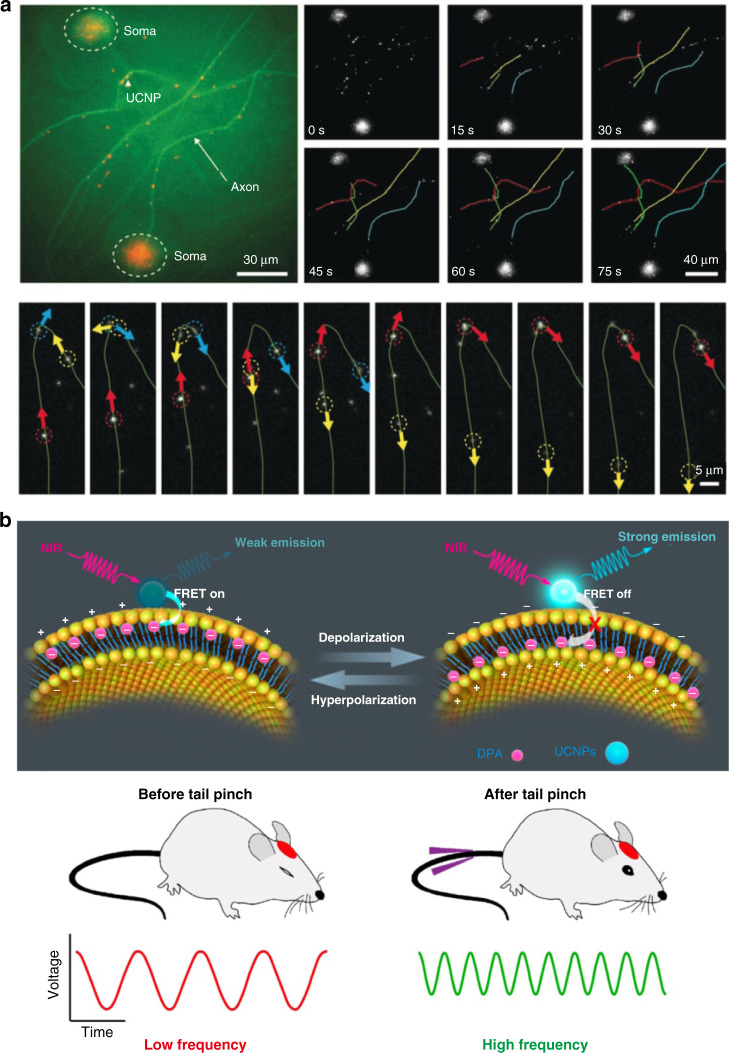
Rare-earth based materials for monitoring the neural activity. **a** UCNPs-mediated visualization of retrograde axonal transport. Reprinted with permission from ref. ^22^. Copyright 2019 Wiley-VCH GmbH. **b** The schematic diagram of the NIR-excited optical voltage sensor to real-time monitor the subthreshold activities in mouse cortical neurons. Reprinted with permission from ref. ^93^. Copyright 2020 American Chemical Society

In addition to brain imaging and disease therapy, RENPs are promising candidates for monitoring brain neuronal activity and diagnosing brain diseases due to the superior luminescent properties. Monitoring neural activity, as well as neurological diseases, is conducive to understanding the pathogenesis and achieving an early diagnosis. Liu et al. used silica-coated NaLuF_4_:Yb^3+^/Er^3+^ UCNPs to realize real-time and continuous tracking of dynein movement inside neurons^22^. UCNPs were endocytosed into the cell and trapped in the endosome. When the membrane receptors on the endosomes bound to cytoplasmic dynein via protein-protein interactions, the dynein would unidirectionally transport the endosomes containing UCNPs to tens of micrometers away at a rate of 1–2 μm s^−1^. The superior photostability allowed tracking the UCNPs movement over 5 min without signal attenuation (Fig. 7a). Therefore, UCNPs-mediated visualization of dynein-driven retrograde axonal transport provided insights into the mechanism of dynein movement, neurological disease pathology and the role of various neural circuits in the brain.

By utilizing FRET strategy between hexanitrodiphenylamine (DPA) and UCNPs, Du’s group designed a NIR-excited optical voltage sensor to real-time monitor the neuronal activity^93^. As shown in Fig. 7b, UCNPs and DPA were immobilized outside the cell membrane and inside the cell membrane, respectively. The absorption wavelength of DPA largely overlapped with the emission spectrum of UCNPs, thus DPA could effectively quench the luminescence of the UCNPs. However, the realization of energy transfer from UCNPs to DPA required a close distance within 10 nm. The location of the negatively charged DPA was affected by the fluctuation of membrane potential, which influenced the distance between the DPA and UCNPs, thereby causing the fluorescent changes of UCNPs. Owing to the superior photostability of UCNPs, the nanosensor could record the changes of the membrane potential with high fidelity for a long time. In addition, they showed the feasibility of the nanosensor for reporting neuronal activity in zebrafish and mice.

### Detect the brain disease

**Fig. 8:**
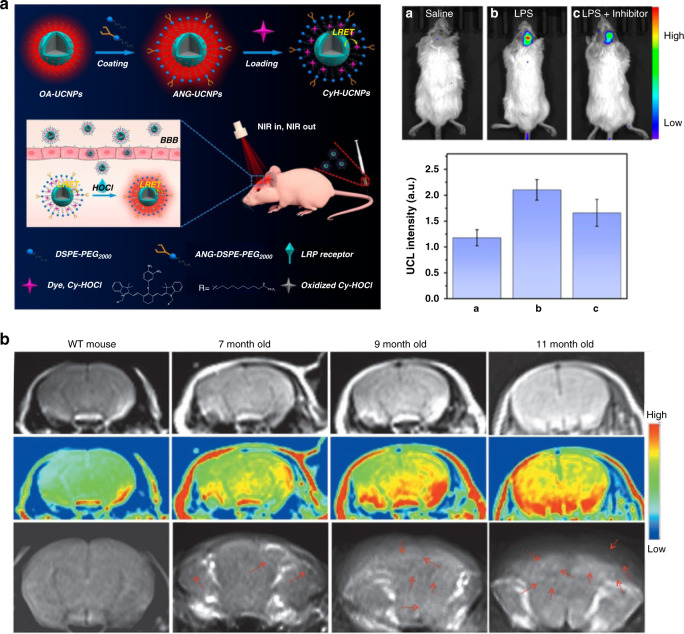
Rare-earth based materials for detecting the brain diseases. **a** HOCl-activated upconversion nanoprobes for visualizing lipopolysaccharide (LPS)-induced neuroinflammation (left). Intravital fluorescence imaging of neuroinflammation in the LPS-induced neuroinflammation model (right). Reprinted with permission from ref. ^94^. Copyright 2020 American Chemical Society. **b** MRI (top), the respective color mapped images (middle) and in vitro images (bottom) of WT and Tg mice brain at different ages after injection of NaGdF_4_:Yb^3+^/Tm^3+^@NaGdF_4_@SiO_2_@F-SLOH. Reprinted with permission from ref. ^24^. Copyright 2020 Wiley-VCH GmbH

In addition to monitoring neural activity, rare-earth based nanoparticles can also be used to prepare fluorescent probes for detecting the brain diseases. Liu et al. designed HOCl-activated upconversion nanoprobes for visualizing neuroinflammation in vivo^94^. Neuroinflammation is induced by activated microglia and astrocytes, accompanied by a substantial production of HOCl^95^. As shown in Fig. 8a, Cy-HOCl dye was assembled with UCNPs via hydrophobic interaction, which acted as energy receptor for UCNPs and recognition unit of HOCl. The CyH-UCNPs nanoprobes could detect HOCl in the LPS-induced neuroinflammation model through monitoring the luminescence intensity of UCNPs. The luminescence intensity in the LPS-treated mice brain showed 1.8-fold enhancement compared with the control group, which suggested that overproduced HOCl could recover the luminescence of UCNPs. After 10 min injection of nanoprobes, the intracerebral fluorescence signal intensity in mice with ischemic stroke was around 1.4 times higher than that in control groups, which enabled the real-time visualization of neuroinflammation. Meanwhile, Wong et al. designed the first Aβ oligomer-selective multimodal (MRI/NIR) contrast agent: NaGdF_4_:Yb^3+^/Tm^3+^@NaGdF_4_@SiO_2_@F-SLOH and achieved detection and imaging of Aβ content in transgenic (Tg) AD mice of different age groups^24^. The formation and deposition of Aβ oligomers are one of the biomarkers representing the early diagnosis of AD^96^. The novel fluorine-substituted cyanine (F-SLOH) in nanosensor exhibited selectively binding affinity to Aβ oligomers and diverse response to different Aβ species^97^. The fluorescence intensity of the nanosensor was different when they bounded with Aβ oligomers, Aβ monomers and fibrils. After 6 h injection of the nanosensor, the cerebrovascular fluorescence intensity of the transgenic AD mice demonstrated distinct age dependence. Older transgenic AD mice displayed a stronger fluorescence signal. Besides, the MRI signal of transgenic AD mice was generally stronger than that of wild-type (WT) mice (Fig. 8b).

### Sensor the ions changes in brain

**Fig. 9:**
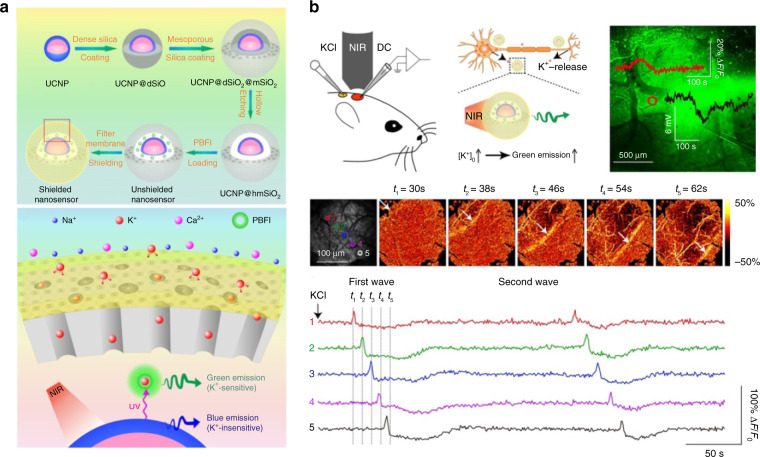
Rare-earth based materials for imaging the ions changes in brain. **a** Schematic diagram of K^+^ ions nanoprobe. **b** Nanoprobe-assisted imaging of extracellular K^+^ waves across the cortical surface in a cortical spreading depression model of mice. Reprinted with permission from ref. ^23^. Copyright 2020 Jianan Liu et al.

The ions changes are closely related to cell metabolism, apoptosis and neurotransmission^98^. The sensor of the ions changes is beneficial for early detection of brain disease. By customized design of rare-earth based materials, the fluctuation of ions concentration in the brain can be reported through monitoring the fluorescence intensity of the UCNPs, thereby realizing in vivo dynamic detection of the ions. Based on the FRET strategy, Chang et al. designed a nanoprobe for rapid detection of Zn^2+^ by loading Zn^2+^-responsive chromophores on the surface of NaYF_4_:Yb/Tm@NaYF_4_ nanoparticles^99^. The UCL of the probe could be quenched by chromophores through the FRET process and restored by the addition of Zn^2+^, which enabled quantitative measuring of Zn^2+^ involved in the pathogenesis of AD^100^. The UCNP-chromophore nanoprobe could detect Zn^2+^ in an aqueous solution with a high sensitivity and rapid response, which shows great potential for imaging of brain tissues with AD. Except for Zn^2+^, the potassium ion (K^+^) channel is believed to be closely associated with seizures^101^. As shown in Fig. 9a, Shi et al. finely designed spherical nanoprobes with a three-layer core-shell structure (UCNPs@K^+^ indicator@K^+^-selective filter membrane) to monitor the variations of K^+^ ions concentration in the central nervous system^23^. The outermost K^+^-selective filter membrane largely enhanced the nanoprobes’ resistance to other cations (Na^+^, Ca^2+^) in vivo, endowing the probes with high K^+^ ions selectivity. And the UV emission of UCNPs in the inner core under NIR light irradiation could excite the K^+^ ions indicator in the middle layer. The nanoprobe could detect K^+^ ions in aqueous media with a detection limit as low as 2.8 × 10^−6^ M and rapid response within 1 s. Ultimately, nanoprobes could achieve dynamic monitoring of K^+^ ions concentrations in the brains of migraine mice and epilepsy zebrafish (Fig. 9b).

## Rare-earth based materials for brain regulation through optogenetics

Optogenetics is an optical technique that uses visible light to activate channel proteins expressed in specific cells to remotely stimulate specific neurons deep in the brain^102^. The optogenetic stimulation of deep brain regions can induce multiple physiological phenomena including dopamine release, seizure inhibition, theta oscillation and memory recall, which could also be used for clinical treatment of neurological disorders^103^. However, the visible light is strongly scattered in the tissues and cannot penetrate deep into brain. In addition, optical fibers are always required and invaded into brain for the optogenetics^104^. UCNPs can transform long-wavelength NIR light into diverse visible light, such as blue light, green light and so on. Benefiting from the unique upconverting fluorescent properties of UCNPs, the UCNP-mediated optogenetics was proposed in 2011, which provides a minimally invasive technique that gets rid of the dependence on optical fibers, avoiding the damage to brain tissue caused by optical fibers^105^. Table 2 lists the recent progress of rare-earth based materials-mediated optogenetics.

**Table 2:** The recent progress of rare-earth based materials-mediated optogenetics

Biological model	Opsins	Absorption wavelength (nm)	Mode	Rare-earth based materials	Year
Cell	C1V1; PsCHR	549; 390–475	Activation	NaYF_4_:Sc/Yb/Er; NaYF_4_:Sc/Yb/Tm@NaYF_4_	2015^106^
	ChR2	470	Activation	NaYF_4_:Yb^3+^/Tm^3+^@NaYF_4_ poly(lactic-co-glycolic acid) scaffffold	2015^107^
	ReaChR	470–630	Activation	IR-806-β-NaYF_4_:Yb^3+^/Er^3+^@β-NaYF_4_:Yb/ poly(methyl methacrylate) matrix	2016^108^
C. elegans	ChR2	475	Activation	NaYF_4_:Yb^3+^/Tm^3+^	2016^109^
Zebrafish	ChR2	480	Activation	NaYF_4_:Yb/Tm/Nd@NaYF_4_:Nd	2017^110^
Mice	ChR2; C1V1	470; 540	Activation	NaYF_4_:Yb/Tm@NaYF_4_/glass micro-optrode; NaYF_4_:Yb/Er@NaYF_4_/glass micro-optrode	2017^111^
	*eNpHR*	550	Inhibition	NaYF_4_@NaYF_4_:Yb/Er@NaYF_4_/glass micro-optrode	2018^112^
	C1V1; ACR1	500-550	Activation; Inhibition	green-emitting lanthanide micro-particles;	2019^113^
	ChR2; Arch	475; 540	Activation; Inhibition	NaYF_4_:Yb/Tm@SiO_2_ UCNPs; NaYF_4_:Yb/Er@SiO_2_ UCNPs	2018^103^

**Fig. 10:**
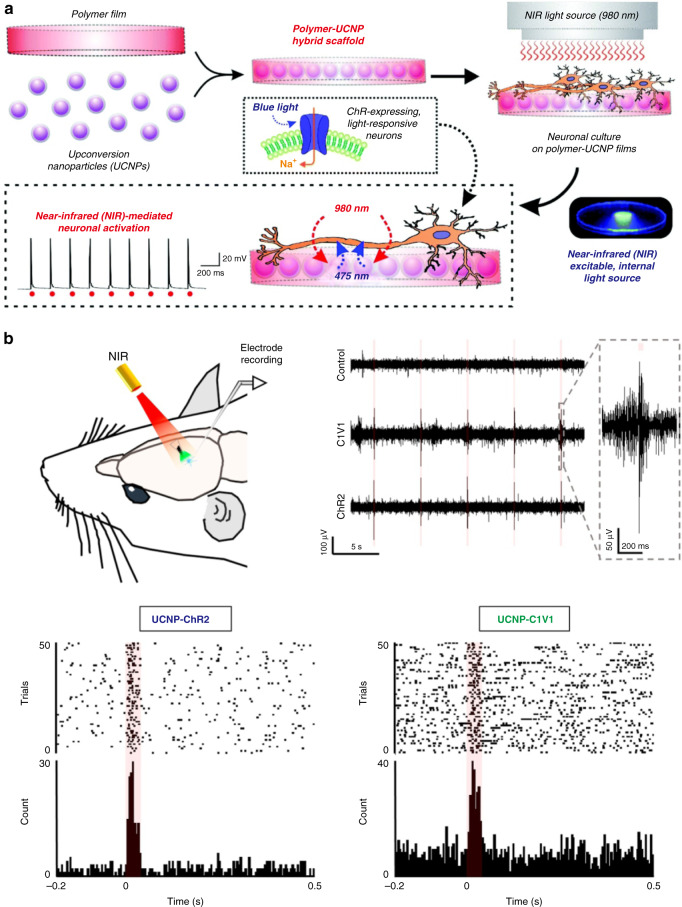
Rare-earth based materials-mediated optogenetics in neuron and mice models. **a** In vitro neuron model of NIR light-induced optogenetics with polymer-UCNPs hybrid scaffolds. The UCNPs emitted blue light to activate ChR2-expressing neurons under NIR irradiation. Reprinted with permission from ref. ^107^. Copyright 2015 The Royal Society of Chemistry. **b** In vivo mice model of UCNPs-mediated optogenetics. The UCNPs-optrode was implanted into a mouse brain and excited through pulsed NIR to record the neuron activities in both ChR2- and C1V1-expressing cells. Reprinted with permission from ref. ^111^. Copyright 2017 Elsevier

In proof-of-concept experiment, Yawo’s group cultured C1V1 and *Platymonas subcordiformis* (PsChR)-expressing neurons on collagen-UCNPs films^106^. Then they performed whole-cell patch-clamp experiments to prove the feasibility of NIR-mediated neuronal activity. The green emission of upconversion substrates under 980 nm irradiation could result in the inward photocurrents in C1V1-expressing neurons. Meanwhile, photocurrents were observed in PsChR-expressing cells when using blue-emitting UCNP-based membrane. Afterwards, Lee et al. prepared inorganic-organic hybrid nanomaterial scaffolds by encapsulating NaYF_4_:Yb^3+^/Tm^3+^@NaYF_4_ UCNPs within the PLGA^107^. The nanomaterials could emit blue light at 470 nm under 980 nm excitation, which could be used for activating channelrhodopsin-2 (ChR2). As shown in Fig. 10a, ChR2-expressing cells were cultured on the PLGA-UCNP hybrid scaffolds and displayed repetitive action potentials at frequencies up to 10 Hz under pulsed NIR light irradiation.

Although the in vitro optogenetics has been realized successfully, it’s challenging to implant these large sized devices into the brain. In order to realize in vivo optogenetics, Shi’s group packaged UCNPs in a micro-optrode and made a fully implantable transducer device (Fig. 10b)^111^. The small size of the UCNPs-optrode (≈100 µm in diameter, <1 mg in weight) enabled the simultaneous implantation of multiple UCNP-electrodes, which was conductive to achieving complex manipulation of brain activity with minor surgical lesions and inflammatory response. In their study, Tm^3+^-doped UCNPs with the emission of 470 nm and Er^3+^-doped UCNPs with the emission of 540 nm were sealed in micro-optrode to activate ChR2 and C1V1 opsins under 980 nm pulses, respectively. In addition, they designed a robotic system to automatically track animal’s head. The combination of upconversion technology and robotic system could achieve effective transcranial nerve stimulation to various regions of the brain, which could be used for behavioral conditioning, locomotion pattern modulation and reflexive learning. For example, more contraversive turning in C1V1 mice (4.68 ± 0.26/min) was induced than that in control groups (1.48 ± 0.17/min) upon NIR irradiation. Furthermore, they encapsulated core-shell-shell UNCPs with emission wavelength around 550 nm in glass micro-optrode to activate enhanced natronomonas halorhodopsin (*eNpHR*) opsin protein for neuronal inhibition^112^. When this UCNPs-based glass micro-optrode was implanted in the rat brain, the neuronal activity was inhibited under NIR irradiation. The system was further applied to perform tetherless deep brain inhibition in freely moving mice. As a result, the total movement distance of the mice was significantly reduced by 49.5%.

**Fig. 11:**
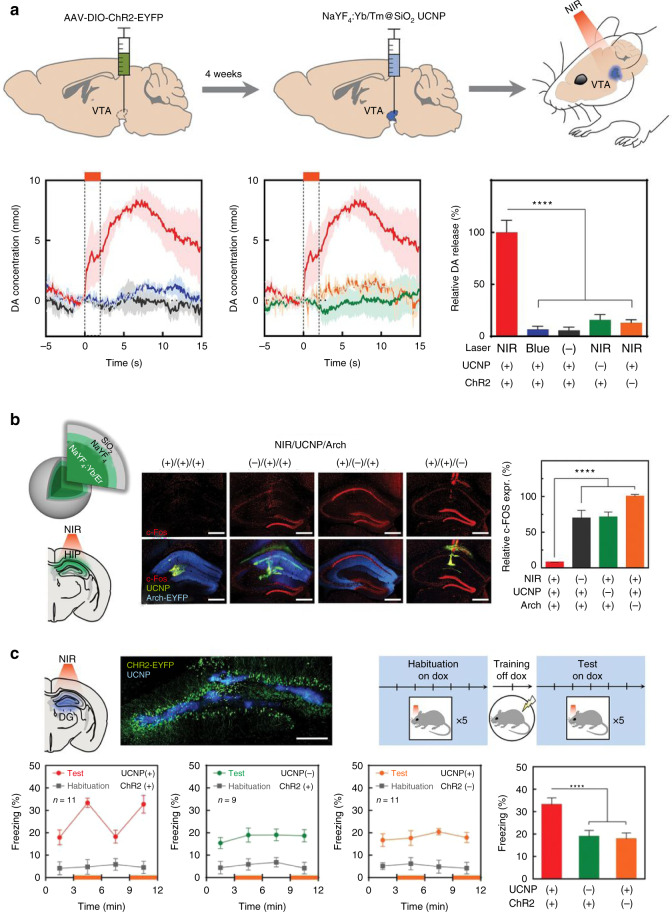
NIR deep-brain stimulation through UCNPs-mediated optogenetics. **a** Transcranial NIR stimulation of VTA dopamine neurons. In the presence of both UCNPs and ChR2, significant release of dopamine can be detected under the stimulation of NIR. **b** Transcranial NIR inhibition of neural activity to silence seizure. **c** Transcranial NIR stimulation of hippocampal engram for memory recall. Reprinted with permission from ref. ^103^. Copyright 2018 American Association for the Advancement of Science

To reduce the brain injury caused by implanting devices, McHugh et al. developed minimally invasive “fiberless” optogenetics technique based on UCNPs^103^. Blue-emitting UCNPs were injected near dopamine neurons in the ventral tegmental area (VTA) of mice to manipulate the release of dopamine (Fig. 11a). In addition, green-emitting UCNPs were also injected in the hippocampus of mice to silence seizure (Fig. 11b). The above processes could be activated under NIR irradiation transcranially. Furthermore, they employed transcranial NIR UCNP-mediated optogenetics to stimulate hippocampal granule cells that related to memory recall, and fear memory in mice could be successfully evoked even two weeks after injection of UCNPs (Fig. 11c).

## Conclusions and outlook

Rare-earth based materials with superior magnetism, unique optical properties, and high X-ray absorption coefficients are superior CAs for MRI, fluorescence imaging, and CT imaging. Notably, the multi-modal imaging based on a single rare-earth based material is also realized, providing more accurate and comprehensive information about brain. Moreover, rare-earth based materials are also effective in the treatment of brain diseases. Radiotherapy, PDT and PTT are applied to treat brain tumors. Luminescent RENPs-based probes are used for early brain diseases diagnosis. UCNPs-mediated wireless optogenetic technique is developed for complex manipulation of brain activity with minor surgical lesions and inflammatory response. In conclusion, rare-earth based materials exhibit bright prospects in the field of integrated diagnosis and therapy. With the continual advancements of synthesis method coupled with the instrument technology, the development of rare-earth based materials for clinical brain disease treatment is highly promising. However, challenges remain in the development of rare-earth based materials:

(1) The fluorescence quantum yield and fluorescence intensity of the RENPs in aqueous solution is still very low. The development of highly robust synthetic methods and efficient structural modulation strategies is required to enhance the optical performance of RENPs.

(2) Most of the reported RENPs are activated by NIR-I region, resulting in inevitable partial consumption of excitation light. Therefore, the development of RENPs with excitation wavelengths located in the NIR-II region is required to obtain clearer and more precise images. In addition, other Ln^3+^ doped RENPs, such as Ho^3+^ and Pr^3+^, are also need to be explored to broaden the available imaging agents.

(3) Overall, NIR-II biomedical fluorescence imaging based on RENPs is still in its infancy. In addition to low fluorescence quantum yield of rare-earth based materials, the lack of high-quality and low-cost charge-coupled device (CCD) detectors is also a major obstacle for NIR-II biomedical luminescence imaging. In addition, the combination setup of NIR-II fluorescence imaging strategy and other imaging strategy is immature and limited until now. The development of longer wavelength fluorescent probes and imaging instruments is urgently required, which will promote the further expansion of multi-modal imaging technology based on the NIR-II region.

(4) Non-invasive delivery of RENPs across the BBB is still a challenge. Currently, focused ultrasound (FUS) assisted technique that harmful to brain is still used to delivery drugs crossing the BBB. Therefore, other BBB-crossing methods including cell penetrating peptide/cells mediated brain delivery and receptor-mediated BBB opening need to be further explored^114^.

(5) Existing clinical CAs still meet the risk of releasing free rare earth ions in complex physiological environment during brain imaging process. In addition, the clearance of RENPs in body also needs to be considered. Thus, it is necessary to develop effective synthesis and assembly strategies to improve the stability, biocompatibility and in vivo clearance of rare-earth based materials.

(6) The technical challenge of implanting UCNPs into the specific deep brain regions with minimal invasion or noninvasion still exists for NIR-activated transcranial optogenetics. The UCNPs are injected stereotactically nowadays, which may cause damage to the brain. Thus, seeking new approaches to deliver UCNPs noninvasively to the specific neurons is urgently needed.
