# Correction: Rare-earth based materials: an effective toolbox for brain imaging, therapy, monitoring and neuromodulation

**DOI:** 10.1038/s41377-022-00922-5

**Published:** 2022-07-16

**Authors:** Zheng Wei, Yawei Liu, Bo Li, Jingjing Li, Shuang Lu, Xiwen Xing, Kai Liu, Fan Wang, Hongjie Zhang

**Affiliations:** 1 State Key Laboratory of Rare Earth Resource Utilization, Changchun Institute of Applied Chemistry, Chinese Academy of Sciences, Changchun, 130022 China; 2 School of Applied Chemistry and Engineering, University of Science and Technology of China, Hefei, 230026 China; 3 Department of Biotechnology, College of Life Science and Technology, Jinan University, Guangzhou, 510632 China; 4 Department of Chemistry, Tsinghua University, Beijing, 100084 China

**Keywords:** Optical materials and structures, Imaging and sensing

Correction to: *Light Science & Applications*

10.1038/s41377-022-00864-y published online 10 June 2022

After publication of this article^1^, it was brought to our attention that the Supplementary Information was attached by mistake, the article is a review article and it doesn’t have Supplementary Information. The original publication has been corrected.
